# Predictive modeling of burnout dimensions based on basic socio-economic determinants in health service managers and support personnel in a resource-limited health center

**DOI:** 10.3389/fpsyt.2024.1519930

**Published:** 2025-01-09

**Authors:** Grey Castro-Tamayo, Mario Hernandez-Tapia, Ivan David Lozada-Martinez, Ivan Portnoy, Jessica Manosalva-Sandoval, Tobías Parodi-Camaño

**Affiliations:** ^1^ Universidad de la Costa, Barranquilla, Colombia; ^2^ Biomedical Scientometrics and Evidence-Based Research Unit, Department of Health Sciences, Universidad de la Costa, Barranquilla, Colombia; ^3^ Universidad de Córdoba, Montería, Colombia

**Keywords:** psychological burnout, risk factors, hospital personnel, resource-limited settings, health services

## Abstract

**Background:**

Burnout is a prevalent condition in the healthcare sector, and although it has been extensively studied among healthcare professionals, less is known about its impact on non-professional workers, particularly in low-resource settings. This study aimed to test a preliminary predictive model based on basic socioeconomic and sociodemographic determinants to predict symptoms of burnout among support personnel and health services managers in a resource-limited health center.

**Methods:**

A prospective cross-sectional study was conducted. Using simple random sampling, symptoms of burnout were surveyed among health service managers and support personnel using the Maslach Burnout Inventory (MBI). Statistical analyses included correlation tests and predictive models using random forest models to identify significant associations and cast predictions.

**Results:**

A total of 76 participants were included. Of these, 34.21% exhibited high levels of emotional exhaustion (EE), 42.11% showed elevated depersonalization (DP), and 7.89% reported low personal accomplishment (PA). Significant negative correlations were observed between household income and the EE and DP dimensions. The predictive models demonstrated acceptable performance in identifying socioeconomic factors associated with burnout, with prediction errors ranging from 7.68% to 20.31%.

**Conclusions:**

Burnout is common among support personnel and health services managers in resource-limited settings, particularly among those with lower incomes. The findings underscore the importance of implementing policies that address both working conditions and economic well-being to mitigate the risk of burnout. More robust predictive models could serve as a valuable tool for early identification and prevention of burnout in this type of setting.

## 1 Introduction

Burnout is a complex occupational condition characterized by the presence of emotional exhaustion (EE), depersonalization (DP), and a reduced sense of personal accomplishment (PA) (1). This condition has become a critical concern in the healthcare sector, especially after the coronavirus disease 2019 (COVID-19) pandemic, which heightened the emotional and workload burdens on healthcare workers (2). Although burnout has been extensively studied among doctors and nurses (2), the prevalence and associated factors of burnout among hospital non-healthcare staff, such as support personnel and health services managers, have received less attention (3).

Studying burnout in hospital non-healthcare staff is essential to better understand the factors that contribute to its development and persistence (4). These workers play vital roles in the day-to-day operations of healthcare institutions, and their well-being is crucial for ensuring a safe and efficient care environment. Unlike healthcare professionals, these workers may have less access to emotional support resources and stress management training, which can make them more vulnerable to burnout (4). This situation may be more intense in low-resource settings and with vulnerabilities of social determinants of health (5). Then, identifying the sociodemographic and economic factors that influence the onset of burnout in this group can facilitate the development of more precise and timely interventions to mitigate its effects (5).

The importance of investigating burnout in resource-limited settings lies in the additional challenges these contexts face (6). Studies conducted in these types of settings have demonstrated that at least eight out of ten healthcare workers experience burnout (7). Work overload, limitations in human and financial resources, and a lack of structural support are factors that can exacerbate occupational stress (6). Moreover, these contexts often have fewer options for institutional intervention and support, making the findings of studies focused on this population particularly valuable for designing cost-effective strategies that can be implemented in similar settings globally. Developing predictive tools based on basic socioeconomic and sociodemographic determinants would be particularly useful, as they could be reproducible in scenarios where these determinants have a significant impact on other social determinants of health and health-related outcomes (8). Given that these types of correlations have not been previously explored in Colombia, this study aimed to test a preliminary predictive model based on basic socioeconomic and sociodemographic determinants to predict symptoms of burnout among support personnel and health services managers in a resource-limited health center.

This study was prepared following the STROBE (Strengthening the reporting of observational studies in epidemiology) recommendations for writing observational studies (9).

## 2 Methods

### 2.1 Study design

This was a prospective cross-sectional study.

### 2.2 Setting and participants

The study was conducted at a secondary healthcare center in Barranquilla, Colombia, which provides local clinical and surgical services related to orthopedics and traumatology. The study population was evaluated at the center during their working hours, between May and September 2024.

A simple random sampling method was used to select support personnel and health service managers related to assistance in health service delivery. This group of workers has been defined and recognized previously as a target group by the World Health Organization (WHO) (10). Inclusion criteria included: 1) age over 18 years; 2) active and regular employment at the center as support personnel and health service managers; and 3) agreement to participate through informed consent. Exclusion criteria were individuals with a history of neuropsychiatric disorders, those currently receiving care from mental health services, or those taking psychiatric medication or any other type of medication with a significant likelihood of causing emotional side effects or adverse reactions.

### 2.3 Variables

The primary outcome, both qualitative and quantitative, was the result of the Maslach Burnout Inventory (MBI) (11). Independent variables included sociodemographic and economic characteristics, as well as the burnout status of the workers.

### 2.4 Data measurement

Basic data were collected through a semi-structured questionnaire administered during face-to-face interviews with each worker. The survey included a scale of educational attainment with the following categories: none, elementary school, middle school, high school, incomplete technical education, technical education, incomplete bachelor’s degree, bachelor’s degree, and postgraduate education. Marital status was classified as single, divorced/separated, domestic partnership, or married. Household income was categorized into three levels: 1–2 minimum wages, 2–3 minimum wages, and more than 3 minimum wages. These classifications have been validated and previously used by local and national governmental institutions (12).

The MBI instrument assesses three dimensions of burnout: EE [9 items], DP [5 items], and PA [8 items]. Each dimension is measured using a 7-point Likert-type frequency response scale (0 = never, 1 = a few times a year or less, 2 = once a month or less, 3 = a few times a month, 4 = once a week, 5 = a few times a week, 6 = every day) (11). Scoring is such that higher scores indicate a greater presence of each construct. Higher scores on the EE and DP subscales suggest a greater burden of burnout symptoms, while lower scores on the PA subscale indicate a greater burden of burnout symptoms.

### 2.5 Statistical analysis

The normality of quantitative variables was tested using the Kolmogorov–Smirnov test. Data were presented as mean ± standard deviation (SD) for continuous variables and median (interquartile, IQR) for skewed variables. Qualitative variables were summarized using frequency and percentages. We conducted a correlation analysis, aiming to unravel the underlying pair-wise interactions between those variables measured by the survey and the burnout-related MBI dimensions. We used Pearson correlations, and hypothesis tests were conducted using Fisher’s Z-transform test to determine their significance with 95% confidence.

Then, we conducted an analysis of variance (ANOVA) for each MBI’s dimension (i.e., EE, DP, and PA) to determine the significance of the effects of the independent variables on these dimensions. For the ANOVA, we included the squared and cubed (independent) variables, as well as their second and third-order interactions, aiming to reveal higher-order, complex interactions that might be missed by the correlation analysis. Consequently, models were created so that EE, DP and PA are modeled in terms of those factors with significant effects on them. This is summarized in the equation:


$$\{\begin{array}{c} EE=f\left(Age,Age^{2},Household\;Income,\;Scholarity,Scholarity^{2}\right)\\ DP=f\left(Household\;Income,\;Scholarity,Scholarity^{2}\right)\\ PA=f\left(Household\;Income^{2},Scholarity^{2},Household\;Income\times Scholarity\right) \end{array}\;$$

For the predictive models, we implemented the regression Random Forest (RF) method, as outlined by Cutler et al. (13) and Speiser et al. (14), to model each MBI’s dimension (EE, DP, and PA). For each outcome, we set the number of trees by iteratively increasing it while monitoring the overall fitting accuracy with the average Mean Absolute Error (MAE) until it does not increase with the number of trees. To evaluate the fitting and prediction accuracy, we randomly split the original dataset into a training and a testing set, comprising 70% and 30% of the samples, respectively. The RF algorithm is trained with the training set, and the testing set is further used to make predictions and evaluate the prediction accuracy with the MAE.

A p-value <0.05 was considered statistically significant. All analyses were performed using the R statistical package (Version 4.3.1) (https://www.r-project.org/).

### 2.6 Ethical statements

This study was approved by the Scientific Committee of the Universidad de la Costa (Minute No 4 – 2024) and was conducted in accordance with the principles of the Helsinki Declaration. Each eligible participant signed an informed consent form.

## 3 Results

A total of 76 workers were included, with a mean age of 37 years (SD 13.25). The majority were women (n=57; 75%), and most had a middle or elementary level of education (74.9%). High levels of EE were observed in 34.21% (n=26) of the participants, while 42.11% (n=32) showed high levels of DP. Only 7.89% (n=6) reported a low sense of PA (**Table 1**).

**Table 1:** Sociodemographic characteristics and burnout outcomes of the population studied (N=76).

Variable	n	%
Age (years), median IQR	37	13.25
Sex		
Male	19	25
Female	57	75
Scholarity		
None	2	2.63
Elementary school	27	35.52
Middle school	30	39.47
High school	5	6.57
Non-completed technician	1	1.31
Technician	1	1.31
Non-completed bachelor	8	10.52
Bachelor	1	1.31
Postgraduate education	1	1.31
Marital Status		
Single	22	28.94
Divorced/separated	28	36.84
Domestic partnership	7	9.21
Married	19	25
Household Income		
1 – 2 minimum wage	64	84.21
2 – 3 minimum wage	10	13.15
3 – 4 minimum wage	2	2.63
Emotional Exhaustion (EE), median IQR	19	18.25
High levels EE	26	34.21
Depersonalization (DP), median IQR	7	15.25
High levels DP	32	42.11
Personal Accomplishment (PA), median IQR	43	7
Low levels PA	6	7.89

DP, Emotional Exhaustion; EE, Depersonalization; PA, Personal Accomplishment.

On average, the workers surveyed experience moderate EE levels (between 19-26), while 34.21% of them experience high EE levels (27-54) and 48.68% experience low EE levels (0-18). Likewise, the average worker exhibits moderate/high DP levels (c.a., 10), with 42.11% of them suffering high DP levels (10-30) and 43.42% showing low DP levels (0-5). As for the PA dimension, the average worker shows high levels of PA (c.a., 41.68), with 71.05% of them exhibiting high PA levels (40-56) and only 7.89% suffering low PA levels (0-33) (**Table 1**).

**Figure 1:**
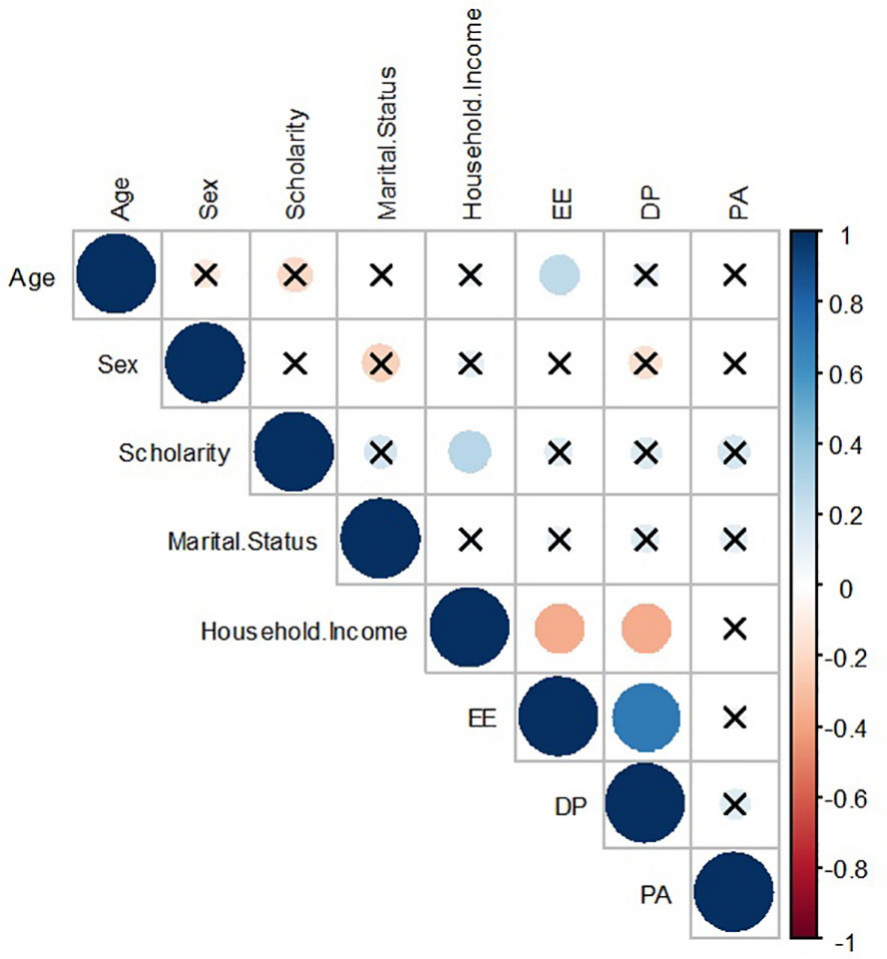
Correlation between basic socioeconomic determinants and burnout dimensions. DP, Emotional Exhaustion; EE, Depersonalization; PA, Personal Accomplishment. Source: authors.

The MBI’s dimensions EE and DP only show significant negative correlations with the household income, while PA shows no significant correlation whatsoever. Although less strong, a positive correlation was also identified between scholarity and household income, and age with EE (**Figure 1**). Specific associations between MBI dimensions and socioeconomic determinants are described in **Table 2**.

**Table 2:** Summary of the analysis of variance between basic socioeconomic determinants and burnout dimensions.

Outcome	Variables with significant effect	p-value
EE	Age	0.00516
	Age^2^	0.01463
	Household Income	9.54e-05
	Scholarity	0.00725
	Scholarity^2^	9.15e-06
DP	Household Income	0.000525
	Scholarity	0.013246
	Scholarity^2^	0.022318
PA	Household Income^2^	0.0417
	Scholarity^2^	0.0663
	Household Income × Scholarity	0.0853

DP, Emotional Exhaustion; EE, Depersonalization; PA, Personal Accomplishment.

Prediction error among the dimensions was identified with a range between 4.301 and 8.241, while a percentage of error between 7.68% and 20.31% was observed (**Table 3**). Then, the proposed models provide a valuable prediction power to determine the MBI’s dimensions as a function of some demographic variables (sex, scholarity, marital status, and household income) with acceptable accuracy.

**Table 3:** Random forest models’ fitting and prediction errors for burnout dimensions.

Outcome	Fitting error (MAE)	Fitting error (as % of span)	Prediction error (MAE)	Prediction error (as % of span)
DP	5.743	16.410%	7.111	20.316%
EE	5.494	8.721%	8.241	13.081%
PA	3.856	6.886%	4.301	7.680%

DP, Emotional Exhaustion; EE, Depersonalization; MAE, Mean Absolute Error; PA, Personal Accomplishment.

## 4 Discussion

The findings of this study reveal that burnout is a prevalent condition among hospital non-healthcare staff at a secondary care center in Colombia, a country with limited resources. The data indicate that 34.21% of participants experienced high levels of EE, while 42.11% exhibited elevated DP levels. Interestingly, only 7.89% reported a low sense of PA, suggesting that, although EE and DP are common, a majority of workers still maintain a positive perception of their performance.

Statistical analysis identified significant correlations between burnout dimensions and specific socioeconomic factors. For instance, a negative correlation was observed between household income and the dimensions of EE and DP, suggesting that workers with lower incomes are more prone to experiencing burnout symptoms. These findings reflect how financial stress can amplify occupational stress and, consequently, the risk of burnout, aligning with previous studies that associate financial strain with mental health issues across various occupations (15, 16). Additionally, the lack of a significant correlation between PA and income underscores the need to evaluate interventions that strengthen the sense of achievement and efficacy at the workplace, irrespective of economic limitations.

In addition to the impact of income, analyzing other socioeconomic factors can provide a more comprehensive understanding of burnout dimensions. For instance, although educational level did not exhibit as strong a correlation as income, a positive trend was observed between lower educational levels and higher scores in EE and DP. These findings suggest that limited access to educational opportunities may exacerbate vulnerability to burnout, particularly in environments where labor and training resources are scarce. Previous studies have documented that workers with lower educational attainment often perceive less control over their work environment and experience greater job dissatisfaction, amplifying their risk of burnout (17). Similarly, job satisfaction emerges as a critical variable interacting with socioeconomic determinants. While this study did not directly measure job satisfaction, existing literature has demonstrated its direct influence on DP and perceptions of PA (18). A lack of recognition and the perception of undervalued roles among support personnel and health service managers may erode the sense of PA, despite the predominantly high PA levels observed in this cohort.

From a theoretical perspective, these findings align with the conservation of resources (COR) theory, which posits that individuals strive to obtain, retain, and protect resources, and that resource loss (e.g., financial strain or lack of social support) is a critical driver of stress and burnout (19). Workers in resource-limited settings, as examined in this study, are likely to face chronic resource loss, which may explain the elevated levels of EE and DP. The significant correlation between low income and these burnout dimensions further supports this framework, as economic instability can be viewed as a continual threat to resource preservation.

Furthermore, the results can also be interpreted through the job demands-resources (JD-R) model, which highlights the imbalance between job demands (e.g., workload and emotional strain) and the availability of job resources (e.g., financial stability and support systems) as a central factor contributing to burnout (20). The high levels of burnout symptoms observed in this study suggest that the job demands on support personnel and health services managers in resource-limited settings exceed their available resources, leaving them vulnerable to chronic stress and emotional exhaustion.

From an evidence-based decision-making perspective, these results emphasize the need to implement psychosocial support and stress management programs for hospital lower-income non-healthcare staff (21). Policies that promote economic stability, such as fair wages and additional benefits, could serve as indirect interventions to mitigate burnout risk (21). Furthermore, the evidence supports the need for training in emotional coping skills that could better equip these workers to manage job demands.

The findings also hold implications for public policy in resource-limited countries. Given the prevalence of burnout among support personnel and health services managers, policy makers should consider policies that address not only working conditions but also the overall well-being of these employees (22). Strategies such as flexible working arrangements, access to mental health services, and recognition, incentives, and support programs may be particularly effective in reducing burnout incidence (21).

The use of predictive models based on RF algorithms to identify determinants of burnout offers an opportunity to apply innovative technologies in managing healthcare human resources (23). These models can assist institutions in identifying at-risk employees based on specific characteristics and implementing preventive measures before burnout symptoms worsen, thereby enhancing intervention effectiveness and optimizing the limited resources available in these settings (23). These findings align with the priorities outlined in the new global health roadmap, which emphasizes the urgent need to generate new, reproducible knowledge that is applicable to resource-limited settings, focusing on health promotion and disease prevention (24).

As limitations, it should be noted that the cross-sectional study design only allows for statistical associations, so results should be interpreted accordingly. Additionally, external validity is limited due to the study being monocentric, analyzing a specific group of hospital non-healthcare staff directly involved in services in a specialized medical area (orthopedics and traumatology). Nonetheless, a key strength is that this analysis provides novel primary data on this often-overlooked workforce segment, as most studies tend to focus on professional healthcare workers. Furthermore, the exploratory approach of using basic, easily accessible data for predictions is notable, as this is commonly the only information available to human resource managers for making occupational health decisions and intervening within their workforce.

## 5 Conclusions

Burnout is common among health services managers and support personnel in resource-limited settings, especially among those with lower incomes. This preliminary predictive model based on basic socioeconomic and sociodemographic determinants can predict with acceptable accuracy burnout symptoms in hospital non-healthcare staff working in secondary centers with limited resources. The findings underscore the importance of implementing policies that address both working conditions and economic well-being to mitigate the risk of burnout on hospital non-healthcare staff. More robust predictive models may offer a valuable tool for early identification and prevention of burnout.

## Data Availability

The raw data supporting the conclusions of this article will be made available by the authors, without undue reservation.
